# Gonorrhœa

**Published:** 1882-08-20

**Authors:** T. H. Logan

**Affiliations:** Ga.


### GONORRHŒA.

By T. H. Logan, M. D., of Ga.

As I have had the benefit of several articles on the treatment of gonorrhœa in your Journal, I wish to give to the profession my experience through the same channel. I have used some of the various remedies laid down for the treatment of gonorrhœa with various results, some have proved successful in my hands, in some cases, while in others they have failed. (What is one man's meat is another's poison). As far as my experience goes in the treatment of gonorrhœa, there is nothing that can truly be called a specific. The treatment I have had most success with and that which seems to be nearer a specific than any other, is as follows:

| | | |
|---|---|---|
| ℞ | Calamine | grs. lxxx, |
| | Pow'd kin | grs. xxx, |
| | Sulph. zinc | grs. x, |
| | Sulph. morphine | grs. viij, |
| | Boiling water | O. j. |

M. Sig. Shake and inject a syringeful every two hours, urinating each time before injecting. The injection should be retained two full minutes, then allowed to escape slowly, so as to leave the sediment in the urethra. The kino must be pulverized and dusted through a fine cloth so as to free it from lumps.

Out of 18 cases treated with the above, there was not a single case in which a cure was not effected inside of 14 days after commencing treatment. Cases seen early in the attack yielded in half that time.

One case especially, that of a young married man of high social standing, consulted me on the 24th of April last; said he had noticed a slight discharge with a burning sensation in the meatus the day before. In my eagerness to try the remedy again, I prescribed the injection only, and impressed upon his mind the importance of using it promptly every two hours; he did so throughout the day and entire night. Next morning he expressed himself well. I gave him a dozen capsules and told him to write to me in two or three days; at the end of that time I received a letter from him stating that not a single symptom of the disease remained. In this case the remedy had a fair trial, and cured the disease in twenty-four hours, though as a general thing it takes a longer time. I use no internal medication until the inflammation has subsided, then a dozen or two capsules will generally suffice to clear up the urethra and remove all remaining traces of inflammation.

The advantage of this treatment over all others I have used is: 1st. The stomach is not crowded with nauseating potions, thereby interrupting digestion. 2d. It is perfectly painless, and not followed by stricture. 3d. The promptness and certainty with which it acts. In none of the cases treated did chordee become a troublesome symptom and not requiring any anodyne treatment, and after a few injections the pain, on urinating, was almost or entirely removed. In chronic cases, when other remedies had failed, it acted like a charm. Occasional coition and horseback exercise did not arrest the progress of cure. Stimulating drinks and articles of diet should be avoided.

This treatment is not original with me, but I have never seen it in any of the medical works. I would like for some of the medical brethren who have never used it to give it a trial and report through the SOUTHERN MEDICAL RECORD, and tell me if I am right in pronouncing it a specific.

