# Mistake

**Published:** 1882-09-20

**Authors:** 


MISTAKE.—The article on Gonorrhœa, in our August number, was written by Dr. T. H. Lyon, and not T. H. Logan, as it was made to appear.

NEW YORK POST GRADUATE MEDICAL SCHOOL.—See the advertisement of this Institution in the present number of our Journal.

BEEF PEPTONOIDS.—See advertisement of this valuable preparation in this No. of Journal.

WE invite attention to Colden's Liquid Beef Tonic in this issue of our Journal.

LISTERINE.—See the advertisement of this valuable article in our Journal.

SEE new advertisement of Anglo Swiss Milk Food.

